# Altered Resting-State Amygdala Functional Connectivity after Real-Time fMRI Emotion Self-Regulation Training

**DOI:** 10.1155/2016/2719895

**Published:** 2016-02-21

**Authors:** Zhonglin Li, Li Tong, Min Guan, Wenjie He, Linyuan Wang, Haibin Bu, Dapeng Shi, Bin Yan

**Affiliations:** ^1^China National Digital Switching System Engineering and Technological Research Center, Zhengzhou, Henan 450000, China; ^2^Department of Radiology, People's Hospital of Zhengzhou University, Zhengzhou, Henan 450000, China

## Abstract

Real-time fMRI neurofeedback (rtfMRI-nf) is a promising tool for enhancing emotion regulation capability of subjects and for the potential alleviation of neuropsychiatric disorders. The amygdala is composed of structurally and functionally distinct nuclei, such as the basolateral amygdala (BLA) and centromedial amygdala (CMA), both of which are involved in emotion processing, generation, and regulation. However, the effect of rtfMRI-nf on the resting-state functional connectivity (rsFC) of BLA and CMA remains to be elucidated. In our study, participants were provided with ongoing information on their emotion states by using real-time multivariate voxel pattern analysis. Results showed that participants presented significantly increased rsFC of BLA and CMA with prefrontal cortex, rostral anterior cingulate cortex, and some others related to emotion after rtfMRI-nf training. The findings provide important evidence for the emotion regulation effectiveness of rtfMRI-nf training and indicate its usefulness as a tool for the self-regulation of emotion.

## 1. Introduction

Emotion regulation plays a vital role in our daily life. Disorder in the ability may affect our work efficiency, induce disharmony in society, and even cause certain psychiatric diseases, including major depressive disorder (MDD), social anxiety disorder (SAD), and autism spectrum disorder [1, 2]. Real-time functional magnetic resonance imaging neurofeedback (rtfMRI-nf) as a promising tool for enhancing emotion regulation capability of subjects and for the potential alleviation of neuropsychiatric disorder symptoms has rapidly developed during recent years [3]. Several studies have demonstrated that individuals could enhance their ability in modulating their own brain activity in structures and networks that are relevant to voluntary emotion processing by using rtfMRI-nf approaches [4–7]. However, the exact neural mechanisms underlying the neurofeedback training effect on emotion regulation remain to be elucidated [3].

To date, the majority of rtfMRI-nf studies have focused on the control of activity in brain areas during task state to demonstrate the effect of training [4, 5, 7, 8]. The resting-state fMRI has been increasingly used to address changes in functional brain connectivity after effective treatments [9]. Resting-state functional connectivity (rsFC) is a highly effective and sensitive method for mapping complex neural circuits speculated to reflect the underlying neuroanatomy [10–12]. In addition, resting-state fMRI is a relatively new modality that potentially overcomes several key limitations of task-stimulated fMRI studies [13]. By comparing rsFC during different stages (before training and after training), the altered FC could be calculated and may indicate the effect of neurofeedback training. Thus, rsFC analysis could be an appropriate method for gaining insight into neural mechanisms underlying neurofeedback training.

According to a cognitive control model of emotion regulation, the neural representation of emotion regulation can be summarized as interactions between the prefrontal cortex (PFC) and anterior cingulate cortex (ACC) systems and their influence on subcortical systems, including the amygdala [14]. The amygdala is a critical region for the generation, expression, and experience of negative emotions, as demonstrated by both animal and human lesion studies [15]. Clinical studies have revealed that resting-state amygdala connectivity is altered in individuals with generalized SAD, social phobia, and MDD [16–18]. Recent functional neuroimaging evidence highlights that the amygdala is composed of structurally and functionally distinct nuclei. The basolateral amygdala (BLA) and centromedial amygdala (CMA) are two major nuclei that play vital roles in emotion processing and generation of behavioral responses [10, 15, 19]. BLA activity correlates extensively with temporal and frontal cortical regions, whereas the predicted activity of CMA is primarily in the hypothalamus, basal forebrain, and brainstem [10, 20]. Thus, the amygdala FC (BLA FC and CMA FC) could be a sensitive biomarker for assessing the effect of rtfMRI-nf on the brain. Research works on psychiatric disease implicated cortex-limbic dysfunction in affect disorders [15]. Several studies have shown that decreased FC between frontal and limbic brain regions is found in major depression patients [21–23]. rsFC could increase after antidepressant treatment [9]. The same result was also found in SAD patients [24]. However, amygdala connectivity at rest in individuals after neurofeedback training remains unclear.

The present investigation aimed to investigate the effect of rtfMRI-nf on amygdala rsFC in a healthy population. Twelve healthy volunteers were trained to self-regulate their emotion states by providing their emotion states (happy or sad) using the real-time multivariate voxel pattern analysis (MVPA) method. The method is suitable for rapidly decoding distributed emotion processes [25]. Resting-state fMRI data were collected before and after rtfMRI-nf training. BLA and CMA were selected as seed regions, and rsFC analysis was performed and compared under different conditions [19, 20]. Given that PFC and ACC exert regulatory control over the amygdala, we hypothesized that increased rsFC between amygdala and PFC or ACC could be observed after rtfMRI-nf training.

## 2. Materials and Methods

### 2.1. Participants

Twelve right-handed healthy volunteers (seven males, mean ± SD age, 23.8 ± 1.4 years) were recruited from China National Digital Switching System Engineering and Technological Research Center. All participants had no history of neurological or psychiatric diseases. The ethics committee of Henan Provincial People's Hospital approved the research protocol. All subjects provided written informed consent to participate in the study and received financial compensation.

### 2.2. Experimental Paradigm

To familiarize the subjects with the experiment procedure, all participants underwent a training session for approximately 30 min at 1 or 2 days prior to scanning. The participants were given detailed instructions about the objective of the study and the experimental paradigm. Each subject was asked to write down three happy and sad autobiographical memories (AMs), respectively. In this experiment, we provided several explicit examples, such as joining a party, obtaining a high score in a course, breaking up with a girlfriend, or losing a beloved dog. The subjects were instructed that they could use happy or sad memories during scanning to induce positive or negative emotions. Then, the subjects were asked to relax to minimize potential motion-related artifacts during scanning.

**Figure 1:**
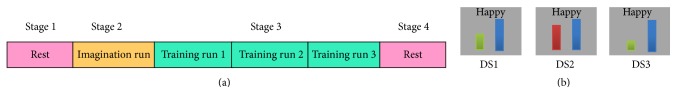
Design of rtfMRI-nf experiment. (a) Experimental procedure consisted of four stages, including two rest runs, one mental imagination run, and three rtfMRI-nf training runs. (b) Three different display screens for rtfMRI-nf procedure during different states of subjects. Display screen 1 (DS1) was presented to the subjects at the beginning of each block. Subjects were instructed to evoke happy autobiographical memories to make themselves feel happy while trying to increase the level of the left bar to a given target level (indicated by the fixed height blue bar). The left red bar in display screen 2 (DS2) indicates the cumulative emotions above the baseline. The left green bar in display screen 3 (DS3) indicates the cumulative emotions below the baseline.

The rtfMRI-nf experiment paradigm consisted of four stages (Figure 1). (1) During the rest runs (stage 1 and stage 4), resting-state fMRI scanning was employed. All subjects were instructed to fixate at the display screen, not to think of anything in particular, and to remain as motionless as possible. A total of 190 functional volumes were obtained. (2) The mental imagination run (stage 2) was block designed and consisted of 172 scans with an altering block of happy and sad imagination that lasted for 20 s and was interleaved with 20 s. During the mental imagination run, each subject was asked to recall AMs, which were prepared before the experiment, as intensely as possible for each type of emotion (e.g., happy or sad). (3) A feature selection mask was generated in two steps. First, a crude selection was made by selecting the top 5% of the voxels based on the *t*-value that excludes visual cortex [25]. Recursive feature elimination is a MVPA method, which considers all voxels in parallel [25]. We used this method for fine-tuning by selecting the top 2% of the remaining voxels. A classification model was trained for each person on the basis of support vector machine (SVM). (4) The rtfMRI-nf training paradigm included three runs (stage 3). The three runs consisted of alternating blocks of rest (six blocks) and train (six blocks) conditions, each lasting 30 s. A total of 192 volumes were carried out per run. During the training run, the present activation pattern (happy versus sad) was decided by trained SVM classifier based on brain volumes which were obtained in real-time. For each volume, the classifier also estimated the distance of a new observation to the separating hyperplane, the classification boundary between conditions. Prior to MRI scanning, all subjects were informed that they would receive information about the activity level of their emotion states, as indicated by the color and height of the bars (Figure 1(b): DS2 and DS3). Subjects were encouraged to try various other AMs if the currently selected AM did not help in raising the red bar during neurofeedback training. The change in bars was calculated on the basis of emotion states and distance of a new observation.

### 2.3. Data Acquisition

Experiments were displayed using Psychopy (http://www.psychopy.org/). All fMRI data were acquired by a 3.0 T GE Discovery MR750 scanner (General Electric, Fairfield, Connecticut, USA) at the Imaging Center of Henan Provincial People's Hospital. A standard 8-channel birdcage head coil was adopted. Head motion was restricted, and scanner noise was diminished using foam pads. A standard GRE-T2^*∗*^ EPI sequence was used to collect functional images with the following parameters: TR (repetition time) = 2000 ms, TE (echo time) = 30 ms, FOV (field of view) = 220 mm, matrix size = 64 × 64, slices = 33, slice thickness = 3.5 mm, and FA (flip angle) = 80°. A high-resolution anatomical scan was acquired using a three-dimensional fast spoiled gradient sequence with the following parameters: TR = 8.2 ms, TE = 3.22 ms, matrix size = 256 × 256, slices = 156, FOV = 240 mm, and FA = 12°.

### 2.4. Preprocessing of fMRI Data

Data preprocessing was conducted using DPARSF (http://www.restfmri.net/), which is based on SPM8 and REST (http://www.restfmri.net/). The 10 initial scans of all experiment runs were discarded because of magnetic equilibration effects that could potentially distort the data. Data preprocessing included the following steps. First, the image data were slice-timing and motion corrected. Second, all functional datasets were transformed into standard Montreal Neurological Institute (MNI) space by linearly registering to the anatomical data and to the MNI152 standard brain. Third, all datasets were smoothed using a 6 mm FWHM Gaussian spatial kernel. Fourth, data were detrended to eliminate the linear trend of time courses and filtered with low frequency fluctuations (0.008–0.01 Hz) [16]. Finally, a set of regressors, including six head motion parameters, white matter mask, cerebrospinal fluid mask, and global mean signal, were regressed out of the EPI time series.

### 2.5. Functional Connectivity Analysis

**Figure 2:**
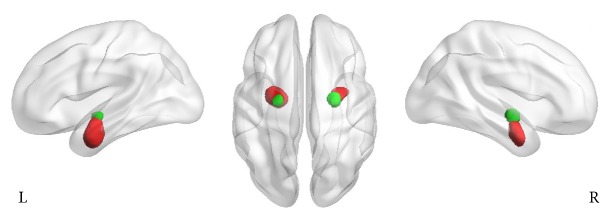
Location of 50% probabilistic masks of basolateral amygdala (BLA, red) and centromedial amygdala (CMA, green). L = left; R = right.

Regions of interest (ROIs) were created using SPM's Anatomy Toolbox [26] with cytoarchitectonically based probability maps of the amygdala (Figure 2), as instantiated in the Juelich Brain Atlas [27]. Only the voxels with a probability of a minimum 50% and belonging to each subdivision (left BLA, right BLA, left CMA, and right CMA) were included. Four ROIs were resampled to 3 × 3 × 3 mm^3^ standard space to enable the extraction of time series from the fMRI data of each subject. The mean time series of all voxels within the ROIs were extracted using DPARSF. The mean time course was then correlated to the time courses of all brain voxels by using Pearson cross correlation. Finally, Fisher's *z* transform analysis was applied to Pearson correlation coefficients to obtain an approximately normal distribution.

Two subjects (one male and one female) were excluded because their six head motion parameters were larger than 1.5 mm of displacement and/or 1.5° of rotation. Separate group statistical analysis was performed for amygdala FC maps by using REST. A one-sample *t*-test was employed to assess the whole-brain rsFC of four ROIs before (pretraining) and after neurofeedback training condition (posttraining) (*p* < 0.01, AlphaSim corrected). To assess the effect of rtfMRI-nf on the brain, we conducted a paired *t*-test. The whole-brain rsFC differences between pretraining and posttraining conditions were compared (*p* < 0.05, AlphaSim corrected).

## 3. Results

### 3.1. rsFC Maps of the Amygdala

**Figure 3:**
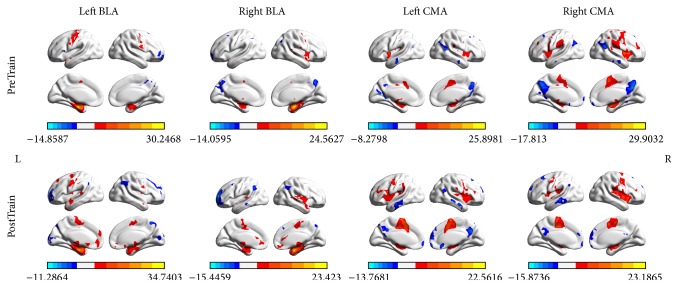
Whole-brain voxelwise rsFC patterns of basolateral amygdala (BLA) and centromedial amygdala (CMA) during preTrain and postTrain conditions. Brain regions with positive correlations are displayed in warm colors, whereas those with negative correlations are displayed in cool colors. Thres: *t* = 3.25, *p* < 0.01, AlphaSim corrected. L = left; R = right.

rsFC maps are shown in Figure 3. The FC patterns for BLA and CMA revealed a frontotemporolimbic network (*t* = 3.25, *p* < 0.01, AlphaSim corrected, minimum 40 voxels). Our results were consistent with the study of Roy et al. (2009) [10].

*BLA*. The left BLA and right BLA seeds showed positive rsFC with bilateral inferior frontal gyrus (IFG, right BLA only), left superior temporal gyrus (STG), middle temporal gyrus (MTG, left for left BLA and bilateral for right BLA), bilateral hippocampus, bilateral parahippocampal, bilateral putamen, left anterior cingulate cortex (right BLA only), insula (left for left BLA and bilateral for right BLA), and bilateral thalamus (right BLA only). Meanwhile, decreased rsFC was found in the bilateral superior frontal gyrus (SFG), middle frontal gyrus (MFG, right for left BLA and left for right BLA), right IFG (left BLA only), bilateral precuneus, and right thalamus (right BLA only).

*CMA*. Increased rsFC with bilateral CMA was found bilaterally in MFG, IFG, STG, hippocampus, parahippocampal, putamen, insula, and thalamus. Decreased rsFC was found in bilateral SFG, MFG (bilateral for right CMA), right STG, MTG (left for left CMA and right for right CMA), ITG (left for left CMA and right for right CMA), bilateral posterior cingulate cortex (PCC), and bilateral precuneus.

### 3.2. Altered Amygdala rsFC after rtfMRI-nf Training

**Figure 4:**
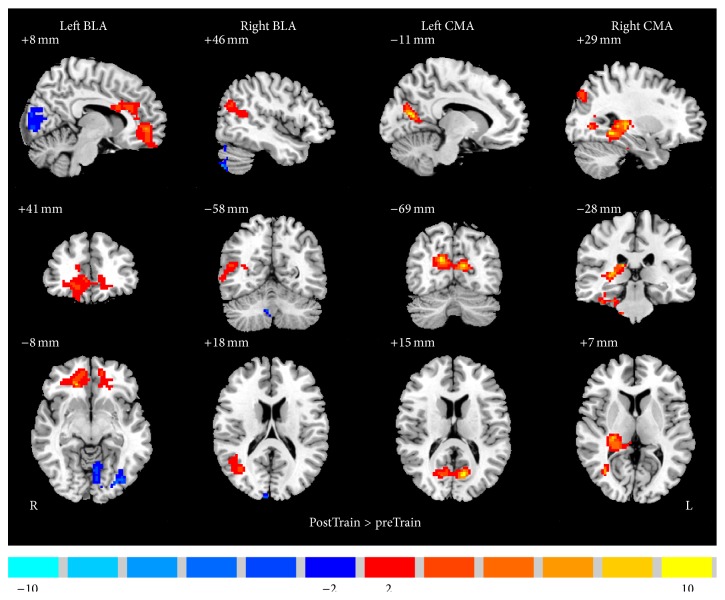
Brain areas that exhibited altered functional connectivity of basolateral amygdala (BLA) and centromedial amygdala (CMA) induced by neurofeedback training. Positive (red) indicates regions in which FC with ROIs (left BLA, right BLA, left CMA, and right CMA) is significantly increased, whereas negative (blue) indicates regions in which FC is significantly decreased (Table 1). Thres: *t* = 2.26, *p* < 0.05, AlphaSim corrected. L = left; R = right.

Results of group rsFC difference analysis are shown in Figure 4. Detailed information on activation centers is specified in Table 1 (*t* = 2.26, *p* < 0.05, AlphaSim corrected, minimum 228 voxels).

*BLA*. Increased left BLA rsFC was found in bilateral rostral ACC (rACC), bilateral medial frontal gyrus (MidFG), bilateral SFG, and right dorsomedial prefrontal cortex (DMPFC) after rtfMRI-nf training. Increased right BLA rsFC was found in left parahippocampal, right MTG, and right STG.

*CMA*. Increased left CMA rsFC was found in bilateral precuneus and bilateral PCC after rtfMRI-nf training. Increased right CMA rsFC was found in right parahippocampal, right hippocampus, right thalamus, right MTG, and right precuneus.

## 4. Discussion

In this study, we investigated the central brain effect of rtfMRI-nf on amygdala rsFC in healthy subjects. The emotion state of participants was provided as feedback signal by real-time MVPA. The results demonstrated that rtfMRI-nf training significantly enhanced rsFC between BLA and PFC, in terms of SFG, MidFG, DMPFC, and rACC, which is consistent with our hypothesis. In addition, increased rsFC was observed between BLA and left parahippocampal, as well as between right MTG and right STG. Increased CMA rsFC was found in bilateral precuneus, bilateral PCC, right parahippocampal, right hippocampus, right thalamus, and right MTG. The altered amygdala rsFC may underlie the central mechanisms of rtfMRI-nf training.

### 4.1. Altered BLA rsFC after rtfMRI-nf Training

PFC and ACC are important parts of cognitive control network, which serves attention-demanding cognitive tasks and emotion regulation [28]. We found enhanced rsFC between BLA and prefrontal cortex (PFC) network, including regions involved in emotion processing and affect regulation, such as the DMPFC, SFG, and MidFG. Interactions between the amygdala and various regions of PFC (especially MPFC) [13, 14, 24] play a fundamental role in the processing and regulation of human emotions. This functional interaction putatively represents the top-down inhibitory control of the amygdala by PFC [29]. In patients with SAD, social phobia, or MDD, PFC appears to be dysfunctional during cognitive-emotional tasks [16, 18]. Hahn et al. [17] found that SAD patients present evidently reduced FC between the left amygdala and the medial orbitofrontal cortex. In addition, Dodhia et al. indicated that OXT enhances the FC between amygdala and frontal cortex (such as MPFC) in patients with generalized SAD. This finding may be attributed to the neural mechanisms for SAD treatment [24]. Moreover, after antidepressant treatment, increased FC between amygdala and PFC was observed in the depressed group [9]. In our study, we demonstrated that rtfMRI-nf training may exert the same effect on amygdala-frontal FC as the drug treatment effect in previous studies.

We also found increased rsFC between left BLA and bilateral rACC. Limbic-cortical-striatal-pallidal-thalamic circuit serves as an important mood regulating circuit (MRC) that involves emotion processing, including generation, regulation, and reaction [35]. In the MRC, the amygdala-ACC circuit plays an important role, and a breakdown in this circuit could potentially decrease the ability of emotion regulation or even cause neuropsychiatric disorders. Several studies have found reduced rsFC between amygdala and rACC in depressed patients, suggesting a decreased regulation effect of the ACC over mood regulating limbic areas [23, 30, 31]. However, increased FC between amygdala and rACC was found in the depressed group after antidepressant treatment [31, 32]. Increased rsFC between the amygdala and ACC may explain mood regulation in rtfMRI-nf training.

### 4.2. Altered CMA rsFC after rtfMRI-nf Training

With regard to CMA, increased rsFC was found in bilateral PCC, bilateral precuneus, right hippocampus, right parahippocampal, right thalamus, and right MTG. The PCC and precuneus are core areas of the default mode network (DMN) that is related to self-referential functions, including mood and reactions to such stimuli [17]. The PCC/precuneus is increasingly recognized to be involved in emotional evaluation and social cognition [33]. Hahn et al. found decreased amygdala FC with PCC/precuneus in SAD [17]. Furthermore, an attenuated amygdala functional connectivity with PCC/precuneus is associated with higher state anxiety symptoms [17]. Therefore, enhanced strength between the amygdala and PCC/precuneus may improve emotion regulation ability to mitigate disease impact. Although the hippocampus/parahippocampal complex and amygdala are related to two independent memory systems with different characteristic functions, they interact in subtle but important manner in emotional situations [34]. In particular, the amygdala could modulate both encoding and storage of hippocampal-dependent memories. In turn, hippocampus also influences amygdala response when emotional stimuli are encountered. The interaction between amygdala and hippocampus/parahippocampal may be strengthened by enhancing the FC between them. The thalamus is related to memory, emotion, and arousal, performing an important function not only in DMN but also in MRC [17, 35]. The circuit between the amygdala and thalamus, together with the orbitofrontal cortex, is responsible for the assessment of threat-related information [17]. Compared with healthy adolescents, MDD adolescents show lower connectivity between the amygdala and several cortical regions (left STG, MTG) during a task condition. Augmentation of FC between amygdala and thalamus or temporal lobe may indicate increased interaction between these regions. These results may also reveal how the rtfMRI-nf training improves the emotion regulation ability of people.

## 5. Limitation

Several limitations should be considered when interpreting these results. First, given the technical complexity, only a small sample size of normal participants was recruited. Thus, the generalizability of the results may be limited. Employing more subjects and depression patients in future studies may be helpful. Second, the real-time MVPA method in the present study was based on spatial pattern of brain activity alone as input in discriminating different emotion states. This method may ignore the temporal pattern or time evolution of brain activity. Hence, better retrained SVM classifiers after each run should be explored to improve the performance. Future studies should involve better retrained SVM classifiers after each run to improve performance.

## 6. Conclusions

In summary, our study demonstrates that rtfMRI-nf training could significantly enhance BLA and CMA rsFC with several regions related to emotion regulation by receiving emotion states. These findings add to our understanding of the neural mechanisms of rtfMRI-nf training. Enhanced rsFC would improve our emotion regulation ability that can yield beneficial outcomes in our everyday lives and numerous social situations. The future of rtfMRI-nf could lead toward several exciting applications in a multitude of neurological and psychiatric disorders.

## Figures and Tables

**Table 1:** Altered functional connectivity induced by neurofeedback training.

Brain regions	Whole cluster size	Cluster size	MNI coordinates			*t* score
			*x*	*y*	*z*	
Seed: L BLA (Post > Pre)						
B rostral anterior cingulate cortex	749	229	9	48	−3	6.74
B medial frontal gyrus		157	33	39	−6	4.26
B superior frontal gyrus		92	21	48	−15	3.32
B ventromedial prefrontal cortex (BA10)		35	6	51	0	3.97
Seed: R BLA (Post > Pre)						
L parahippocampal gyrus	315	54	−30	−18	−30	7.24
R middle temporal gyrus	311	139	36	−63	15	5.27
R superior temporal gyrus		56	51	−58	15	4.60
Seed: L CMA (Post > Pre)						
B precuneus	341	129	−12	−69	15	6.91
B posterior cingulate cortex		75	−9	−69	12	4.67
Seed: R CMA (Post > Pre)						
R parahippocampal gyrus	445	110	30	−45	−9	7.03
R hippocampus		67	30	−36	3	4.49
R thalamus		39	15	−24	15	6.39
R middle temporal gyrus	315	78	48	−69	27	4.55
R precuneus		69	36	−75	36	3.37

L = left; R = right; B = bilateral; BA = Brodmann Area; Pre = pretraining; Post = posttraining. Thres:  *t* = 2.26, *p* < 0.05, AlphaSim corrected.
